# High expression level of *ERBB2* and efficacy of trastuzumab deruxtecan in desmoplastic small round cell tumour: a monocentric case series report

**DOI:** 10.1016/j.esmoop.2025.104133

**Published:** 2025-02-07

**Authors:** M. Brahmi, H. Vanacker, A. Dufresne, V. Isnardi, M. Dupont, A. Meurgey, M. Karanian, P. Meeus, M.-P. Sunyach, F. Tirode, J.-Y. Blay

**Affiliations:** 1 Centre Léon Bérard & Université Claude Bernard Lyon I, Lyon, France; 2 Cancer Research Center of Lyon, INSERM U1052—CNRS UMR5286, Lyon, France

**Keywords:** desmoplastic small round cell tumours, human epidermal growth factor receptor 2, antibody–drug conjugate, trastuzumab deruxtecan

## Abstract

**Background:**

Desmoplastic small round cell tumours (DSRCTs) represent an ultra-rare subtype of soft tissue sarcoma characterized by a recurrent *EWSR1::WT1* oncogenic translocation. Considered as an extremely aggressive cancer, the prognosis remains poor with a median overall survival not exceeding 24-36 months and a 5-year survival <10%.

**Patients and methods:**

We analysed *ERBB2/*human epidermal growth factor receptor 2 (*HER2*) expression levels in a series of 13 DSRCT patients, using whole-exome RNA sequencing on formalin-fixed paraffin-embedded samples from a local biopathological database. In addition, a retrospective case series describes the clinical outcome of three successive DSRCT patients treated with trastuzumab deruxtecan (T-DXd).

**Results:**

The gene expression analysis demonstrated a consistent high RNA expression level of *ERBB2* in DSRCT, with elevated levels [>5 log2(transcripts per million + 1)] across all samples of the cohort and the expression level was the highest compared with all other sarcoma subtypes. In addition to these results, T-DXd showed a marked activity in all three DSRCT patients who presented with metastatic disease refractory to previous standard chemotherapy. So far, the treatment has been overall well tolerated and is currently pursued in the three patients (duration of response >3 months for all three), which warrants additional investigation.

**Conclusions:**

This case series presents a major information, suggesting that HER2 is a therapeutic target in DSRCT and T-DXd might represent a novel therapeutic option. Those results require to be rapidly shared with the scientific community and confirmed in a prospective clinical trial in this context of very poor prognosis disease and urgent unmet need.

## Highlights

• Within soft tissue sarcomas, DSRCTs are an ultra-rare subtype.
• DSRCT is characterized by its clinical aggressiveness, its resistance to chemotherapy and its overall poor prognosis.
• Using RNA sequencing, DSRCTs are characterized by a consistent ERBB2 high expression level.
• This work provides preliminary efficacy data of T-DXd for DSRCT.

## Introduction

Soft tissue sarcomas are rare and heterogeneous malignant tumours accounting for <1% of all adult malignant tumours and 20% of childhood malignancies.^1^ Over the last decade, major advances in molecular characterization have driven further refinements in the fifth World Health Organization Classification of Soft Tissue and Bone Tumours published in 2020.^2^ As of today, >150 distinct mesenchymal tumours are described. Within this group of rare tumours, some subtypes are even rarer, accounting for <1% of all sarcomas.^3^ The management of these ultra-rare sarcomas (URSs) is challenging, especially in advanced stages. Indeed, clinical research dedicated to rare cancers is complicated by recruitment difficulties and faced with the lack of interest of drug developers.

Among these so-called URSs, desmoplastic small round cell tumour (DSRCT) has an estimated incidence between 0.2 and 0.5 new cases per million per year. It is characterized by a recurrent oncogenic translocation t(11;22)(p13;q12) that fuses EWS RNA binding protein 1 gene (*EWSR1*) to Wilms tumour gene 1 (*WT1*).^4^ It affects mostly adolescents and young adults, with a male predominance. Considered as an extremely aggressive cancer, >90% of patients present synchronous peritoneal metastases and ∼50% present synchronous extraperitoneal metastases. Despite a multimodal treatment approach including systemic multiagent chemotherapy, debulking surgery and possibly hyperthermic intraperitoneal chemotherapy and/or whole abdominopelvic radiotherapy, the prognosis remains poor with a median overall survival not exceeding 24-36 months and a 5-year survival <10%.^5^^,^^6^

Trastuzumab deruxtecan (T-DXd) is an antibody–drug conjugate (ADC) composed of a humanized anti-human epidermal growth factor receptor 2 (HER2) monoclonal antibody, a cleavable tetrapeptide-based linker and a cytotoxic topoisomerase I inhibitor as payload. In HER2-positive and HER2-low breast cancers, HER-positive gastric or gastroesophageal junction cancers and HER2-mutant non-small-cell lung cancer, T-DXd has become a Food and Drug Administration (FDA)-approved standard of care. Furthermore, it has recently been approved by the FDA as a histology-agnostic treatment for HER2-overexpressing solid tumours, defined as HER2 immunohistochemistry (IHC) 3+.^7^ However, in the DESTINY-PanTumor02 study that supported the recent histology-agnostic FDA approval,^8^ there were no sarcomas. Also, the expression of ADC targets including *ERBB2*/HER2 in sarcomas remains underreported, preventing the development of ADCs such as T-DXd in this rare and heterogeneous family of tumours, whereas their use could represent a major therapeutic opportunity.

Therefore, we first investigated the expression level of *ERBB2* in a retrospective series of DSRCT, using whole-exome RNA sequencing (WERS) carried out on routine molecular testing. Then, we explored the clinical significance, reporting a case series of patients with treatment-refractory metastatic DSRCT treated with T-DXd.

## Patients and methods

### Histopathology, RNA sequencing and gene expression analyses

This study was carried out at the Centre Léon Bérard as a retrospective translational research programme since 2017. All tumour samples were obtained from the pathology department and consisted of formalin-fixed paraffin-embedded (FFPE) tissue samples at initial diagnosis, obtained by biopsy or surgical excision to establish the diagnosis of the disease. Each case was reviewed by a pathological expert of sarcoma from the Réseau de Référence en Pathologie des Sarcomes (RRePS) network. When confronted with a suspected diagnosis of DSRCT based on morphological and immunohistochemical criteria, pathologists turn routinely to WERS technique to identify potential oncogenic alterations including the canonical fusion *EWSR1::WT1* and therefore to establish the final diagnosis. Integrative WERS and transcriptomic and gene expression analysis were carried out as previously described.^9^

### Clinical case selection and description

The study population consisted of adult patients, with a diagnosis of metastatic refractory DSRCT (who had progressed on at least two lines of therapy), with available information on tumour characteristics, administered therapies and outcomes, and with no opposition from the patients for data use. Patients were included if they received T-DXd, regardless of the line of treatment. The decision of treatment with T-DXd was made following a multidisciplinary sarcoma tumour board (MTB) recommendation, based on the patient’s history and results of molecular tumour testing. Patients’ and tumour characteristics and follow-up were collected from medical records. The following data were collected: birthdate, gender, date of primary tumour diagnosis, tumour status at diagnosis (localized/locally advanced/metastatic), date of progression, date of last follow-up, status at last follow-up (alive without disease/alive with disease/dead), *EWSR1::WT1* fusion results, other relevant molecular, treatment administration (name, start date, stop date, best response, reason for discontinuation, progression date). Follow-up was calculated starting from the date of the diagnosis.

### Study approval

The study was approved by the local study committee and by the national authorities according to the MR004 procedure (https://www.cnil.fr/fr/declaration/methodologie-de-reference-04-recherches-nimpliquant-pas-la-personne-humaine-etudes-et-evaluations-dans-le-domaine-de-la-sante) in October 2024.

## Results

### WERS analyses and expression level of ERBB2 in DSRCT

**Figure 1:**
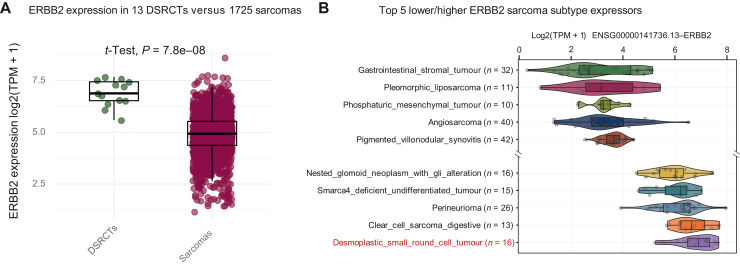
**Schematic representation of*****ERBB2*****expression levelwithwhole-exome RNA sequencing.** (A) Relative expression of *ERBB2* mRNA in 13 DSRCTs versus 1725 various subtypes of sarcomas. (B) Top 5 lower/higher *ERBB2*-expressing sarcoma subtypes. DSRCT, desmoplastic small round cell tumour.

We analysed the expression level of ERBB2 within a series of 1725 mesenchymal tumours including 13 DSRCTs. All 13 DSRCTs harboured the *EWSR1::WT1* oncogenic fusion and the unsupervised analyses on the expression profiles revealed the 13 cases as a specific and biologically homogeneous cluster among the other tumours. In addition, the gene expression analysis demonstrated a high RNA expression level of *ERBB2* in DSRCT, with elevated levels [>5 log2(transcripts per million + 1)] across all samples of the cohort and the expression level was the highest compared with all other sarcoma subtypes (Figure 1A and B).

### Baseline characteristics and outcome

The case series included three patients with metastatic refractory DRSCTs receiving T-DXd (off-label use decision after MTB recommendation). Patient 1 (P1) is a 38-year-old man who presented with a metastatic disease at diagnosis (peritoneal, liver and lung). He showed a partial response with vincristine, doxorubicin, cyclophosphamide, ifosfamide and etoposide (VDC/IE) that lasted 7 months and then a primary resistance to successive systemic therapies including temozolomide irinotecan (TEMIRI), pazopanib and trabectedin. Patient 2 (P2) is a 28-year-old man who presented with a metastatic disease at diagnosis (peritoneal and liver). He showed a 5-month stable disease with VDC/IE and then a primary resistance to TEMIRI. Patient 3 (P3) is a 32-year-old man who presented with a metastatic disease at diagnosis (peritoneal, liver, lung and bone). He showed a partial response with VDC/IE followed by a combination of cyclophosphamide and vinblastine as maintenance therapy. After 15 months, he presented with a progressive disease that showed primary resistance to TEMIRI.

**Figure 2:**
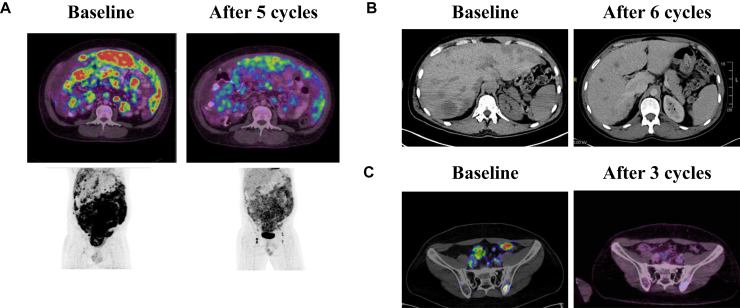
**Radiological evaluation of response to treatment.** (A) 18F-fluorodeoxyglucose-positron emission tomography (FDG-PET) in P1 at baseline and after five cycles of trastuzumab deruxtecan, displaying partial metabolic response on peritoneal sarcomatosis. (B) CT in P2 at baseline and after six cycles of trastuzumab deruxtecan, displaying partial response with both regression and disappearance of liver lesions. (C) FDG-PET in P3 at baseline after three cycles of trastuzumab deruxtecan, displaying partial major metabolic response with both regression of a bone lesion and disappearance of a peritoneal lesion. CT, computed tomography; P, patient.

Intravenous injections of T-DXd 5.4 mg/kg for 3 weeks were started in July 2024 for all three patients. P1 baseline symptoms before T-DXd start included pain, ascites and declining performance status.^3^ He presented with a marked clinical response after three cycles, with complete opioid discontinuation (versus 70 mg twice daily of extended-release morphine) and marked reduction of ascites. The clinical improvement translated into radiological partial response. After five cycles, he presented a significant partial metabolic response on sarcomatosis (Figure 2A). P2 baseline symptoms before T-DXd start included pain, dyspnoea with the need of supplemental oxygen and declining performance status.^3^ He also presented with a marked clinical response after three cycles, with a major decrease in opioid dosage (allowing the discontinuation of morphine patient-controlled analgesia pump) and oxygen therapy weaning. On the computed tomography scan, he presented after six cycles with both regression and disappearance of liver lesions (Figure 2B) and complete disappearance of pulmonary lesions. P3 suffered mainly from pain due to bone metastasis. A superficial 15-mm bone metastasis of the skull was no longer palpable after only one cycle. He also presented with both regression and disappearance of peritoneal and/or bone lesions (Figure 2C).

So far, the treatment has been overall well tolerated, with no dose reduction, interruption or discontinuation. T-DXd is currently pursued in the three patients (duration of response >3 months for all three).

## Discussion

This work reports on the expression level of *ERBB2* in a series of 13 DSRCTs using RNA sequencing in clinical routine practice. It was found consistently high, even the highest compared with other sarcoma histological subtypes. Importantly, a high and statistically significant correlation between RNA sequencing on FFPE materials and IHC measurements for HER2/ERBB2 has been previously demonstrated.^10^ In addition to the high expression levels of *ERBB2*, this work provides preliminary data on T-DXd efficacy in metastatic refractory DSRCT patients, with three successive metabolic partial responses reported. Otherwise, a recent article also described the high expression level of *ERBB2* in DSRCT, using clinical samples and patient-derived xenografts (PDXs), and reported responses to T-DXd in DSRCT PDX, cell line, and organoid models.^11^

In the last few years, research into the molecular aspects of sarcomas has increased greatly.^12^ Unfortunately, beyond the oncogenic fusion, no actionable genomic alterations have been identified in DSRCT as of today, preventing from the development of targeted therapies.^13^ Therefore, conventional chemotherapy remains the standard treatment of DSRCT, though those diseases are known to be quite chemoresistant, especially beyond the first-line treatment.^14^ Otherwise, the ADC track might be relevant among diseases where chemotherapy remains the systemic treatment of reference. In this perspective, the increasingly routine use of RNA sequencing facilitates translational research and evaluation of ADC targets in sarcomas.

The EWSR1–WT1 fusion chimeric protein promotes DSRCT tumourigenesis, acting as an aberrant transcription factor of downstream targets.^15^ DSRCT is therefore dependent on the activity of this oncogenic fusion. While it is likely that the activity of T-DXd in DSRCT results from the consistent high level of expression of *ERBB2*, the correlation between EWS–WT1 transcriptional activity and ERBB2 remains elusive as of today.

DSRCT is a highly lethal cancer and the activity of second- and later-line treatment is very limited, with no much recent improvement in management.^5^^,^^16^ One of the reasons for this is the rarity of the disease, which impacts the feasibility of clinical trials, but also impact the pharmaceutical industry’s interest. But despite these challenges, the conduction of dedicated trials is crucial. The observation of three radiological responses, consistent with a recent congress presentation,^17^ presents valuable information to the scientific community regarding the management of this sarcoma subtype. Also, importantly, the evaluation of the efficacy of T-DXd in a larger series of DSRCT patients is warranted to validate those results and it should therefore be explored in a prospective clinical trial (a phase II study is scheduled).

In conclusion, DSRCT shows consistently a high expression level of *ERBB2* and T-DXd seems highly and rapidly efficient, regardless of the line of treatment, leading to a major improvement of symptoms and quality of life in these young patients with refractory metastatic DSRCT. These findings suggest that T-DXd could be a valuable therapeutic option to be evaluated in DSRCT and need eagerly to be confirmed in a prospective trial in this context of urgent unmet need.
